# Adenoid Cystic Carcinoma Metastasized to Colon

**DOI:** 10.7759/cureus.2085

**Published:** 2018-01-18

**Authors:** Patricia Guzman Rojas, Jignesh Parikh, Priya Vishnubhotla, Jeannette Vergeli-Rojas

**Affiliations:** 1 Internal Medicine, UCF College of Medicine; 2 Pathology, Orlando VA Medical Center; 3 Medicine, Hematology-Oncology, Orlando VA Medical Center; 4 Gastroenterology, Orlando VA Medical Center

**Keywords:** adenoid cystic carcinoma, liver metastases, colon, lung mass

## Abstract

Adenoid cystic carcinoma (ACC) is an infrequent cause of malignancy that accounts for 1% of all tumors of the oral and maxillofacial region.

We present a 59-year-old woman with a past medical history of adenoid cystic carcinoma of the left salivary gland treated with radiation and thoracotomy due to lung metastasis. Years after the onset of diagnosis, she presented with nonspecific gastrointestinal symptoms. For this reason, an abdominal computed tomography (CT) scan was done, revealing a liver mass in the right lobe, involving segments eight and five, concerning for malignancy. A colonoscopy was indicated for screening purposes, showing a large polyp that was biopsied. A histopathologic examination of the colon polyp and a liver biopsy was compatible with ACC metastatic carcinoma.

We report this case to highlight an unusual location of metastatic ACC. Furthermore, there is no case report in the literature where colon metastasis has been described.

## Introduction

Adenoid cystic carcinoma (ACC) is an infrequent cause of malignancy, accounting for 1% of all tumors from the oral and maxillofacial region [1]. It is known that this type of malignancy can arise from any mucous gland from the hard palate, tongue, and other areas [2].

## Case presentation

We present a 59-year-old woman with a past medical history of adenoid cystic carcinoma of the left salivary gland treated with surgery followed by radiation. Seven years later, the patient underwent an osteoradionecrosis and mandibulectomy with reconstruction. At that time, a staging computed tomography (CT) scan that showed bilateral lung metastasis with a biopsy and a confirmatory diagnosis of adenoid cystic carcinoma of the lung. Therefore, she underwent a bilateral thoracotomy, without radiation or chemotherapy.

**Figure 1:**
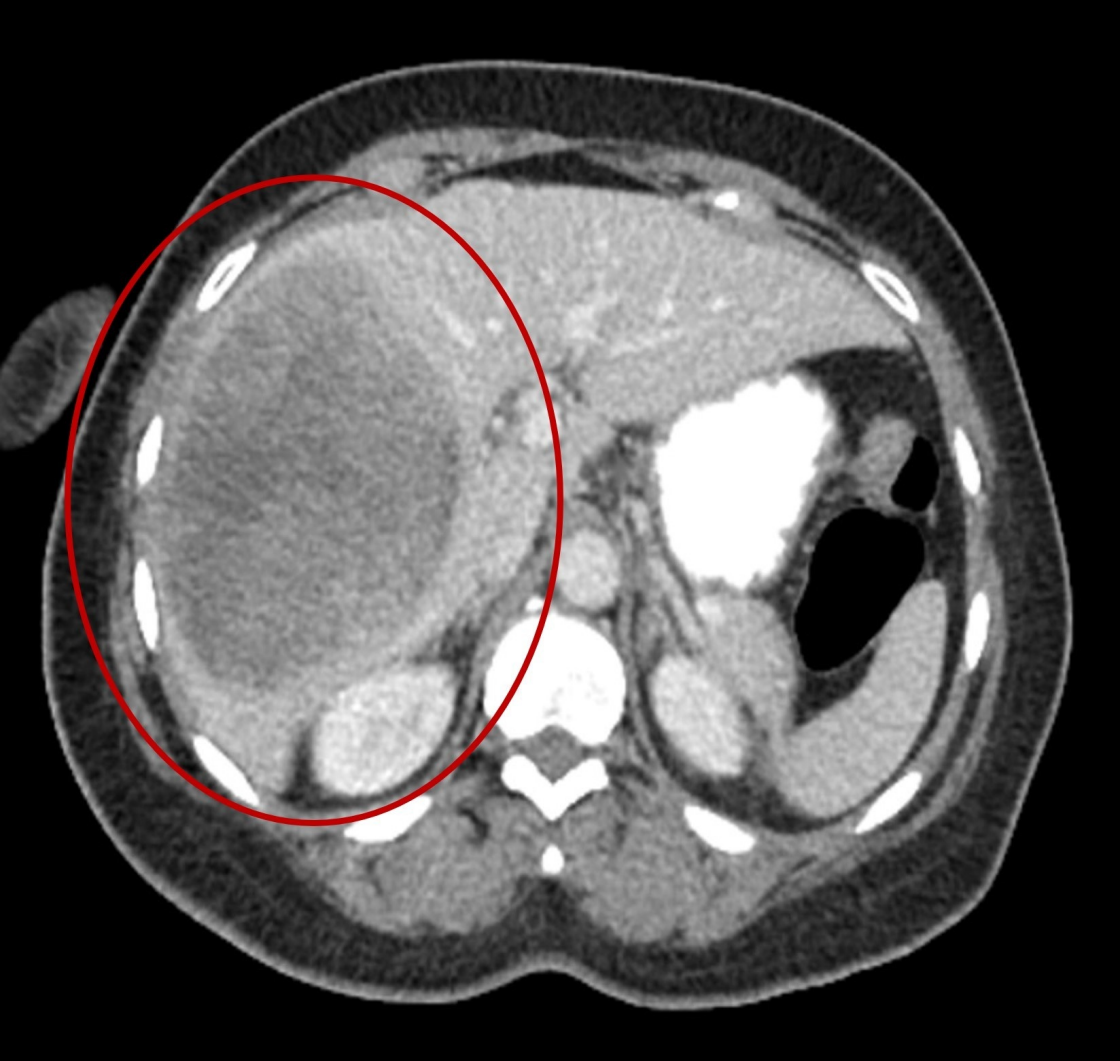
CT of the abdomen: 11 cm mass in the right lobe. CT: computed tomography

**Figure 2:**
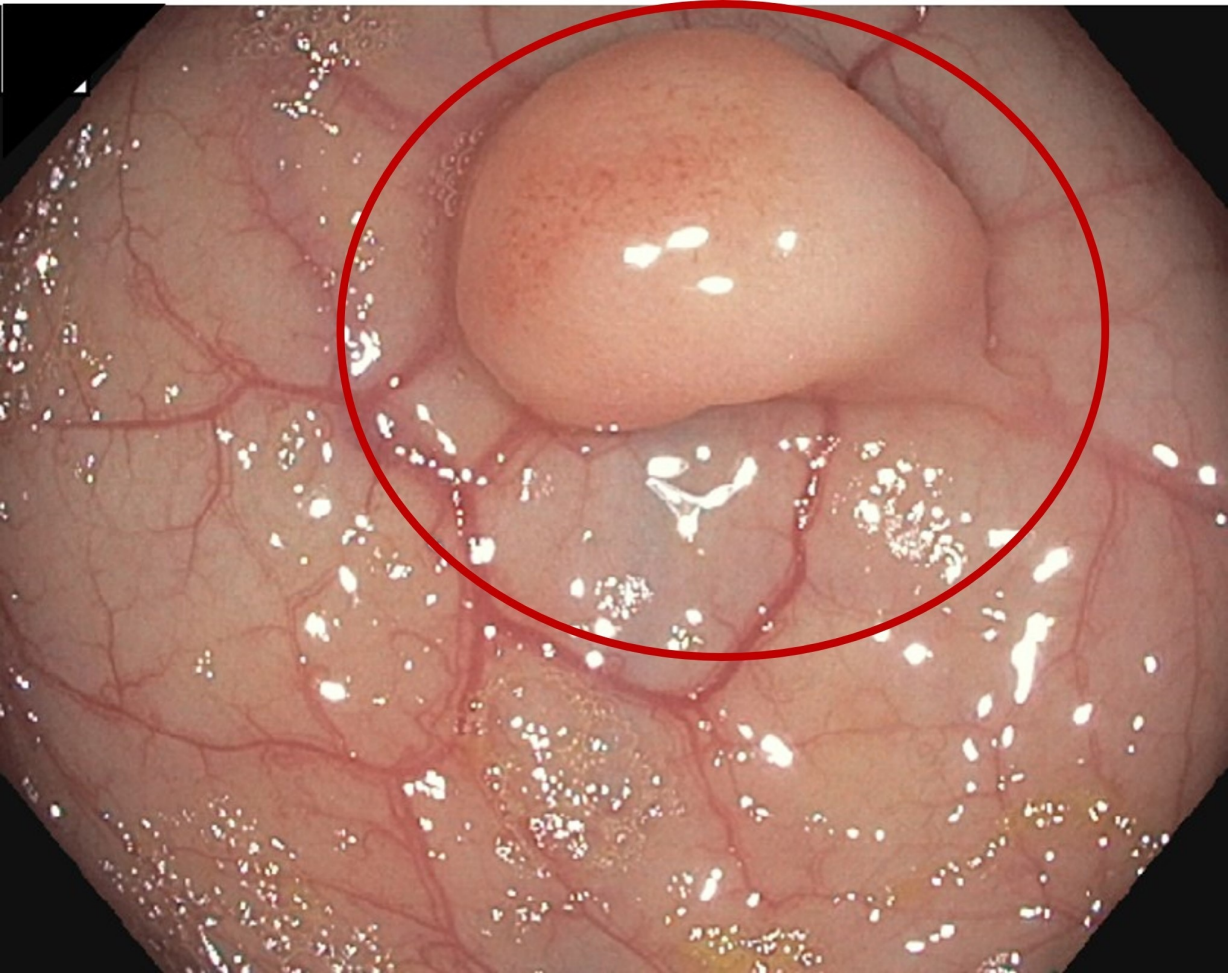
Colonoscopy: 1.2-1.5 cm polyp found at 45 cm from the anus.

**Figure 3:**
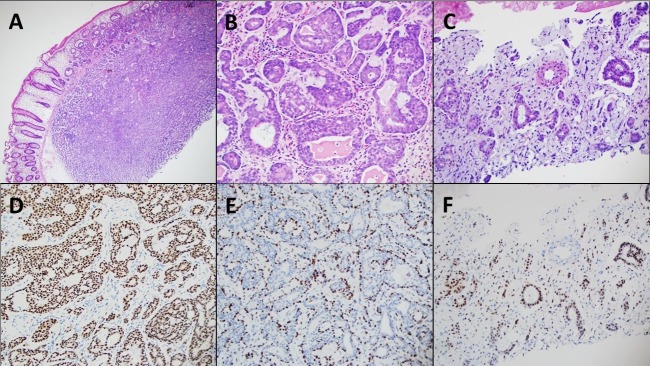
Hematoxylin and eosin stained sections show the area of metastatic carcinoma in colonic (A and B) and liver biopsies (Figure C). Areas of classic ACC are seen in the colonic biopsy (Figure B). SOX 10 is expressed both in luminal and abluminal cells (Figure E, colon; Figure G, liver). P63 shows nuclear reactivity in abluminal cells (Figure F). ACC: adenoid cystic carcinoma

Years after this diagnosis, she presented to urgent care with nonspecific symptoms of nausea, vomiting, and diarrhea. During the evaluation, a CT scan was done, which revealed a liver mass (Figure 1). A magnetic resonance imaging (MRI) scan confirmed an 11-cm mass in the right lobe, involving segments eight and five, concerning for malignancy. An upper endoscopy (EGD)/colonoscopy was scheduled as clearance prior to possible partial hepatectomy. The EGD showed no abnormalities; however, the colonoscopy showed a large, 1.2-1.5 cm hard, sessile polyp at 45 cm from the anus (Figure 2). No other major abnormalities were found. A histopathologic examination of the colonic polyp and a liver biopsy were compatible with metastatic carcinoma (Figure 3). The colonic tumor showed areas of classic ACC; however, the liver tumor did not have the fully developed typical morphology of ACC. Immunohistochemical studies for CK7, SOX-10, p63, S100, BCL2, and CD117 were consistent with metastatic ACC in both colon and liver tumors. After the diagnosis of metastatic ACC, the patient was referred to oncology for palliative treatment. Furthermore, the patient declined any further therapy and was established with hospice care.

## Discussion

Salivary gland carcinoma is a rare malignancy that has multiple histological subtypes, with ACC making up only 10% of them [3]. Even though the progression of ACC is slow, it has a poor outcome. Furthermore, it is already established that this malignancy has frequent recurrences and a late onset distant metastasis. The organs most frequently involved are the lungs, bones, brain, and liver. The most commonly involved intraoral site is the palate, but this could also arise from the tongue [2].

As was stated by Gondivkar, et al., the epidemiology of this malignancy shows an increased frequency in women in the fifth and sixth decades of life; moreover, it is rarely found in the population younger than 20 years old [2]. The histopathology of this type of cancer represents a mixture of myoepithelial and ductal cells [2]. There are three characteristics patterns: cribriform, tubular, and solid.

The treatment is based on surgery, radiotherapy, chemotherapy, and combined therapy. Early surgical intervention is the preferred modality for localized ACC; however, a strategy to prevent relapses is adding radiotherapy [1]. Nonetheless, the presence of distant metastases incentivizes a palliative approach, with radiation and/or adjuvant chemotherapy. Conventional chemotherapy regimens, such as cisplatin and fluorouracil (5-FU) or cisplatin, doxorubicin, cyclophosphamide (CAP), are still the first-line therapy.

As is expected, the literature describes an advanced tumor stage as a poor prognostic factor; nevertheless, some authors identify the histological subtype (trabecular and solid) as a high degree of malignancy [4-5]. Particularly, these subtypes have been associated with recurrences and early distant metastasis.

According to the NCCN guidelines [6], surveillance is based on histoy and physical every one to three months, during the first year; every two to six months during the second year; every four to eight months during years three and five and every 12 months after the first five years. Routine annual imaging can be indicated in areas difficult to visualize on exam.  It is important to mention, that the only recommended distant imaging is a chest CT in patients with smoking history.

There are few case reports where patients with ACC present with metastasis to the liver [7-9]; however, there is no literature yet on cases where a metastasis is found in the colon. On the other hand, Harish et al. [7] reported a patient that presented metastasis even after a surgical excision was done, like ours.

## Conclusions

We report this case to highlight an unusual location of metastatic ACC. Furthermore, there is no case report in the literature where colon metastasis has been described. We would like physicians to be aware of the possibility of distant and late metastases of this type of malignancy.
